# Granulomatous pyoderma preceding chronic recurrent multifocal osteomyelitis triggered by vaccinations in a two-year-old boy: a case report

**DOI:** 10.1186/1752-1947-4-325

**Published:** 2010-10-18

**Authors:** Neslihan Karaca, Guzide Aksu, Can Ozturk, Nesrin Gulez, Necil Kutukculer

**Affiliations:** 1 Ege University School of Medicine, Department of Pediatric Immunology, Izmir, Turkey; 2 SB Tepecik Egitim Hastanesi, Department of Pediatrics, Izmir, Turkey

## Abstract

**Introduction:**

Chronic recurrent multifocal osteomyelitis is a rare, systemic, aseptic, inflammatory disorder that involves different sites. Pathogenesis of chronic recurrent multifocal osteomyelitis is currently unknown.

**Case presentation:**

A two-year-old Caucasian boy, diagnosed with chronic recurrent multifocal osteomyelitis with granulomatous pyoderma following routine vaccinations is presented for the first time in the literature.

**Conclusion:**

We conclude that antigen exposures might have provoked this inflammatory condition for our case. Skin and/or bone lesions following vaccinations should raise suspicion of an inflammatory response such as chronic recurrent multifocal osteomyelitis only after thorough evaluation for chronic infection, autoimmune, immunodeficiency or vasculitic diseases.

## Introduction

Chronic recurrent multifocal osteomyelitis (CRMO) is a rare, systemic, noninfectious, inflammatory disorder that is characterised by recurrent, nonsuppurative, multiple osteolytic bone lesions. It accounts for 2% to 5% of all osteomyelitis cases [1,2].

It mainly affects metaphyses of the long bones with repetitive exacerbations and spontaneous remissions and is frequently associated with a cutaneous inflammatory condition such as pustulosis palmoplantaris, Sweet syndrome, psoriasis and pyoderma gangrenosum [3,4].

We hereby present a case of chronic recurrent multifocal osteomyelitis initially presenting as granulomatous pyoderma following routine vaccinations.

## Case presentation

A two-year-old Caucasian boy, the first child of non-consanguineous healthy parents, presented with history of recurrent skin lesions. These lesions occurred after BCG (Bacillus Calmette-Guerin) and DTP (diphteria, tetanus and pertussis) vaccinations at the age of two months and after each hepatitis B vaccination thereafter. Skin lesions were initially papular, then vesicular with purulent exudate, progressing to multiple ulcers and draining sinuses spreading from the injection site.

**Figure 1:**
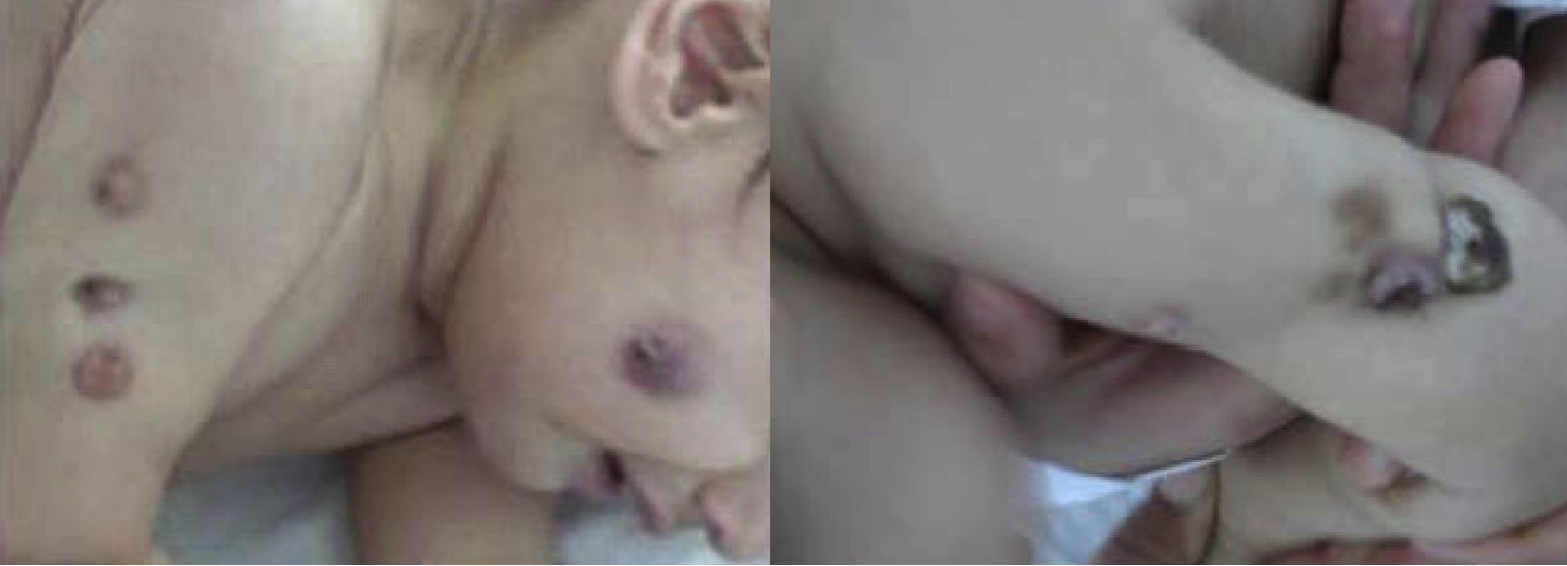
**Violeceous, tender, superfically ulcerated plaques on right deltoid area, right cheek and right forearm**.

On admission, skin examination revealed violaceous, tender; 5-7 mm sized superficial ulcerations with draining sinuses on right forearm, left deltoid area and right cheek (Figure 1). There were no constitutional symptoms or other abnormality in physical examination. Laboratory results were as follows; white blood cell count 9220/mL with 56% polymorph nuclear cells, 40% lymphocytes, 4% monocytes on peripheral smear, hemoglobin 11.4 g/dL, platelets 426.000/mL, erythrocyte sedimentation rate (ESR) was 24 mm/h and the C-reactive protein was 0.43 mg/dL. X-rays of humerus, radius and ulna were normal. Possibilities of combined immunodeficiency, hyper IgM syndrome types I/III, chronic granulomatous disease, IL-12/interferon-gamma pathway defects were excluded: Immunoglobulin G-M-A serum concentrations, lymphocyte subsets, expression of CD40 on B cells, CD40 ligand on active T cells, complement levels (C3, C4), adenosine deaminase level, phagoburst test, expression of CD119 (interferon-gamma receptor) and IL-12 receptor β-I on lymphocytes were within normal ranges. Functional and genetic studies related with IL-12 β1 and IFN-γ receptors were normal. Histopathology of skin biopsy specimen showed 'noncaseating granuloma'. Regarding the initial appearance of the lesions after BCG vaccination and histopathology, scrofuloderma was considered. However, acid-fast bacillus smears and initial cultures for nonspecific bacteria and mycobacteria were negative. The patient was empirically treated with isoniazid 5 mg/kg, rifampicin 10 mg/kg and pyrazinamide 25 mg/kg and skin lesions improved gradually.

**Figure 2:**
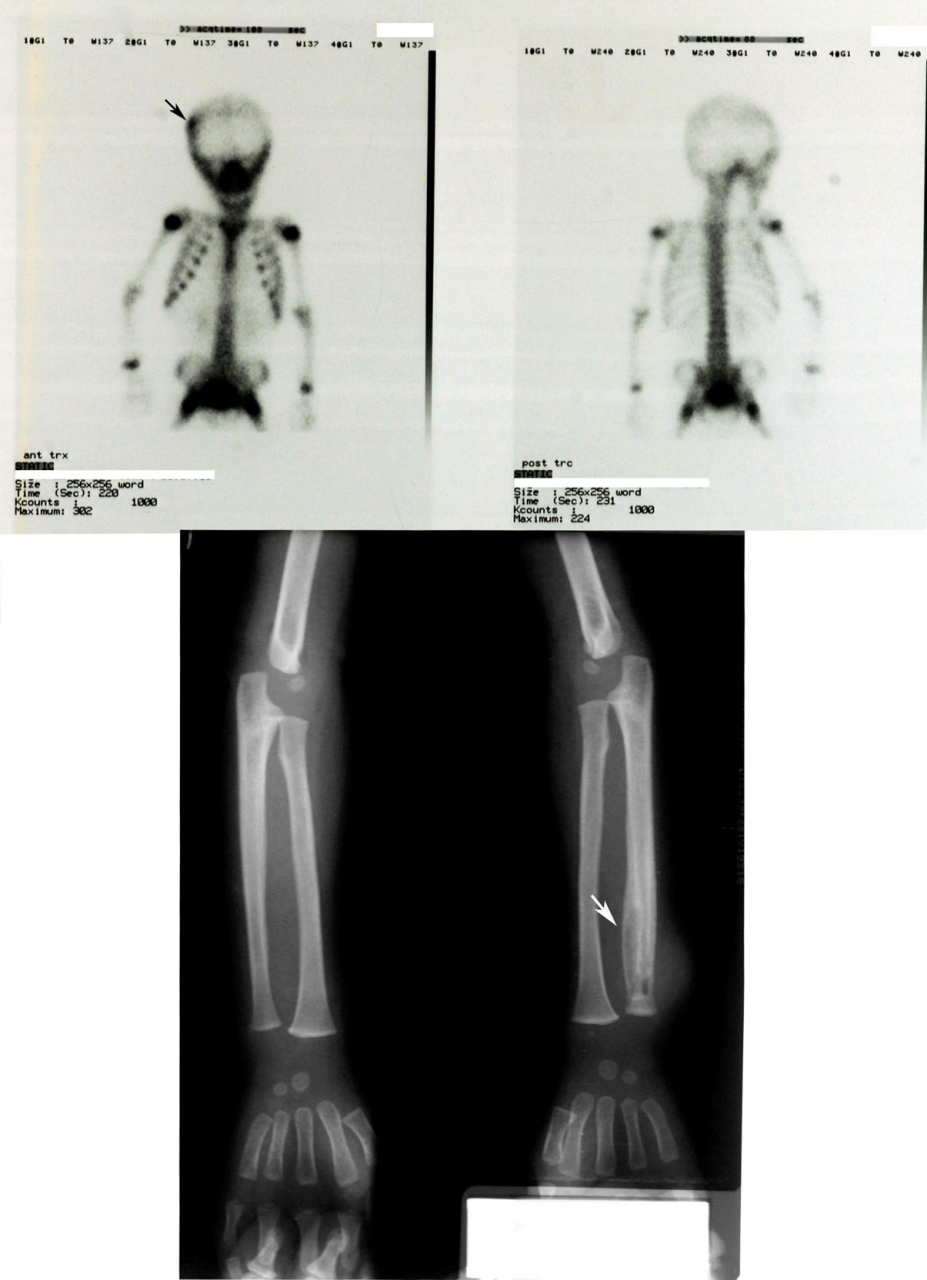
**Bone Tc 99m MDP scintigraphy (a) showing increased activity on right frontoparietal bone: (b) plain radiograph of the patient showing osteolytic lesions in distal metaphyseal region of the radius**.

Five months after initiation of anti-mycobacterial treatment, an elevation in ESR to 74 mm per hour was obtained. On physical examination, he did not have new skin lesions. Nonspecific and mycobacterial cultures of blood and urine, peripheral and bone marrow aspiration smears, Mantoux skin test, serological investigations for Brucella and Salmonella, abdominal ultrasonography were normal. Rheumatic and auto-inflammatory diseases including sarcoidosis and vasculitis were searched out; HLA-B27 antigen, anti-nuclear antibody, antineutrophilic cytoplasmic antibody and rheumatoid factor were negative. Genetic analyses for 'Familial Mediterranean Fever', 'Tumor Necrosis Factor Receptor-Associated Periodic Syndrome' and IL-1 receptor defects were performed with negative results except IL-1 receptor antagonist intron 2 variable tandem repeat polymorphism (IL-1RN-1/1). Ciprofloxacin was added to anti-mycobacterial treatment and ESR decreased to normal (18 mm per hour). After a period of two months with no complaints, he developed new purulent skin lesions on the left forearm and left medial malleolus. Increased activity was seen on right frontoparietal bone with bone Tc 99m MDP scintigraphy; X-ray of the distal metaphyseal region of the radius revealed osteolytic lesions (Figure 2). Bone biopsy was planned but parental consent was not given for the procedure. The patient was treated with intravenous teicoplanin for three weeks. Cultures of abscess material, taken before antibiotic treatment, for bacteria (including acid-fast bacilli) and fungi yielded negative results. During the following three months, he was given anti-mycobacterial treatment including isoniazid and rifampicin for nine months and pyrazinamid for five months. After this period, the patient was readmitted with pain and swelling on both ankles. The ankles were swollen and warm. X-ray of the left distal tibia showed 'osteolytic lesions surrounded by reactive hyperostosis' consistent with chronic osteomyelitis. He was diagnosed as CRMO based on four clinical exacerbations and repeatedly negative cultures. Prednisolone (0.8 mg/kg/day) treatment was started and all other medications were stopped. X-ray of tubular bones showed disappearance of all osteolytic lesions two months after the initiation of corticosteroid therapy. He was complaint-free during the following 18 months.

According to the initial presentation with multiform skin lesions affecting left deltoid area and right cheek just after BCG vaccination at the age of two months and the biopsy findings of the skin lesions, our patient raised the suspicion of scrofuloderma and empirical anti-mycobacterial treatment was given [5]. Mycobacterial disease was not supported by skin tests, lesional smears or repeated cultures before treatment. Possible inherited defects in the defence against mycobacterial infections such as IL-12 β and IFN-γ receptor deficiencies were ruled out with functional and genetic investigations. On admission, X-rays of left humerus, radius and ulna were normal.

During follow-up, he had new skin lesions as well as recurrent, sterile, multifocal, osteolytic bone lesions with reactive hyperostosis interpreted as chronic osteomyelitis. As broad-spectrum antibiotics and anti-mycobacterial treatment were not effective, he was treated by corticosteroids and complete remission was then obtained. The prompt response to steroid rather than antibiotic treatment raised the possibility of an inflammatory condition rather than an immunodeficiency. Collectively, we diagnosed the patient as having CRMO at 29 months of age with a history of disease course of more then three months and failure to cultivate a micro-organism.

## Discussion

The etiology of CRMO remains unknown. Rheumatic disease, bacterial subacute osteomyelitis and malignancy are the main differential diagnoses. These were excluded in our case. The histopathology of bone lesions is variable. Chronic lesions demonstrate a predominance of lymphocytes with the occasional presence of plasma cells. Non-caseating granulomatous foci occasionally coexist [6,7]. The diagnosis of CRMO remains a challenge. Schultz *et al. *[8] suggested that the disease can be diagnosed in the presence of a prolonged course more than three months, evidence of bone inflammation, negative bone cultures by an open bone biopsy and the presence of multiple bone lesions. The impossibility to perform a bone biopsy, due to lack of parental consent, represents a relevant limitation to the correct interpretation of the clinical and pathological findings observed. Recurrent, multifocal, sterile osteomyelitis in X-rays and bone scintigraphy findings with negative cultures supported the diagnosis of CRMO for our case.

CRMO primarily affects bone but is often accompanied by chronic inflammatory neutrophilic dermatoses such as palmoplantar pustulosis, psoriasis, severe acne, Sweet syndrome, pyoderma gangrenosum or superficial granulomatous pyoderma [4,9]. In our case, it was preceded with granulomatous pyoderma. Little is known about the simultaneous presence of chronic musculoskeletal inflammation and skin disorders. CRMO has been considered to be the pediatric variant of SAPHO (synovitis, acne, pustulosis, hyperostosis and osteitis) syndrome [7]. In a report presenting ten cases with SAPHO syndrome during childhood, the ages at onset ranged from 2.9 to 13.5 years [7]. The age that the first lesions appeared in our patient was two months, which is extremely young for disease onset. The increased prevalence of HLA-B27, sacroiliitis, inflammatory bowel disease and psoriasis in patients with SAPHO syndrome has led it to be classified as a spondyloarthropathy [10]. Our case was HLA-B27 negative and lacked these rheumatologic manifestations.

In most patients, cultures from bone lesions are sterile. *Propionibacterium acnes *has been found in the affected area, in a few cases. There is no response to antibiotics. Some authors suppose *P. acnes *as a trigger in the pathogenesis of the disease [11]. However, these bacteria might also be contaminants during biopsy. As all clinical symptoms preceded various vaccinations in our patient, it can be speculated that antigen exposures might have triggered this inflammatory condition.

Although most reported cases of CRMO are sporadic, there is evidence for a genetic component to its etiology. There is an autosomal recessive syndromic form of CRMO (Majeed syndrome) which is caused by mutations in LPIN2 [12,13]. In addition, mice with a mutation on chromosome 18 develop a syndrome resembling human CRMO, suggesting a possible genetic predisposition [14]. There is also evidence to suggest that the bony inflammation in CRMO is a result of an aberrant immune response directed against bone [15]. In our case, the bony symptoms have improved after treatment of antiinflammatory drugs.

CRMO is generally treated with nonsteroidal anti-inflammatory drugs, corticosteroids, analgesics and antibiotic therapy is not recommended [2]. In our patient, skin lesions and multifocal osteomyelitis responded well to oral prednisolone treatment.

## Conclusion

Clinicians should be aware of CRMO, because it typically occurs during childhood and should be included in the differential diagnosis of patients with signs and symptoms of recurrent osteomyelitis and granulomatous pyoderma to avoid prolonged antibiotic treatment.

Granulomatous pyoderma with CRMO triggered by vaccinations was not previously reported. This novel association may serve to enlighten the currently unknown pathogenesis of CRMO.

## Competing interests

The authors declare that they have no competing interests.

## Consent

Written informed consent was obtained from the parents of the patient for publication of this case report and accompanying images. A copy of the written consent is available for review by the Editor-in-Chief of this journal.

## Authors' contributions

All authors have analysed and interpreted the patient data regarding the auto-inflammatory disease. All authors have read and approved the final manuscript.
